# Electroacupuncture at *Jing-jiaji* points for neck pain caused by cervical spondylosis: a study protocol for a randomized controlled pilot trial

**DOI:** 10.1186/1745-6215-14-360

**Published:** 2013-10-29

**Authors:** Zhong-ren Sun, Jin-huan Yue, Qin-hong Zhang

**Affiliations:** 1 Department of Acupuncture and Moxibustion, First Affiliated Hospital of Heilongjiang University of Traditional Chinese Medicine, Harbin 150040, PR China; 2 Department of Acupuncture and Moxibustion, Second Affiliated Hospital of Heilongjiang University of Traditional Chinese Medicine, Harbin 150040, PR China; 3 Department of Acupuncture and Moxibustion, College of Acupuncture and Moxibustion, Heilongjiang University of Chinese Medicine, 24 Heiping Road, Xiangfang District, Harbin, Heilongjiang Province 150040, China

**Keywords:** Electroacupuncture, Acupuncture, Cervical spondylosis, Randomized controlled trial

## Abstract

**Background:**

Neck pain caused by cervical spondylosis (CS) has become one of the most common health problems around the world. Electroacupuncture (EA) has been employed to relieve CS neck pain, but there is limited clinical evidence for its effectiveness.

**Methods/Design:**

This study consists of a randomized controlled trial (RCT) with two parallel arms: an acupuncture group and an EA group. Both groups will receive acupuncture at *Jing-jiaji* points for 30 minutes each time, for five sessions per week for a total of 20 sessions during this four-week period. In addition, the EA group will be connected with EA apparatus. The following outcome measurements will be used in examination of subjects: the Northwick Park Neck Pain Questionnaire (NPQ), McGill Pain Questionnaire (MPQ), and Short-Form 36 (SF-36) scale. All these outcomes will be examined at the start of the study, at the end of the second week, at four weeks after randomization, and one and three months after treatment cessation respectively.

**Discussion:**

This study aims to assess the efficacy of EA, compared with acupuncture intervention at *Jing-jiaji* points for the CS neck pain.

**Trial registration:**

Chinese Clinical Trials Register: ChiCTR-TRC-13003422.

## Background

Cervical spondylosis (CS) is defined as an age-related chronic disc degeneration, which is caused by unspecified degenerative changes of the muscles, tendons, joints, and bones of the neck and shoulder [1]. Its etiology involves several factors, such as poor posture, anxiety, depression, neck strain, and sporting or occupational activities [2]. The main symptoms of CS are neck pain and stiffness, even radiating to the shoulders, and sometimes accompanied by numbness and radicular pain in the arms and fingers.

The prevalence and incidence of neck pain caused by CS have been reported in several studies. For example, the overall prevalence of neck pain rates vary from 0.4% to 86.8% (mean 23.1%) and the incidence of neck pain rates fluctuate from 10.4% to 21.3% in a high-risk population (office and computer workers) [3]. In the UK, about two-thirds of adults suffer from the experience of neck pain at some time in their lives [4]. A household survey was conducted in Hong Kong showed that the prevalence of CS neck pain to be 15% to 17%, and the lifetime prevalence to be 30% to 50% [5].

Conventional medical treatments for CS neck are limited by their modest effectiveness [6]. These kinds of treatments include administration of NSAID drugs [7], muscle relaxants [8], physiotherapy [9], analgesics [10], stress management [11], and so on [12].

Traditional Chinese medicine interventions, such as Chinese herbal medicine [13], acupuncture [14], massage [15], cupping therapy [16], and neck exercises [17] have been widely used for the management of CS neck pain. Of those, acupuncture is one of the most popular measures.

In China, the evidence-based potentiality for effective use of acupuncture in the treatment of CS neck pain has been suggested [1,14,18-22]. However, the current level of evidence is poor due to the scarce good quality studies, small sample size, short duration, rare follow-up, and variation in the composition of acupuncture. In addition, two rigorously designed RCT protocols of acupuncture for CS neck pain have recently been published [23,24]. However, the efficacy of EA for CS neck pain has not been reported specifically. Considering these methodological flaws, we will conduct a trial to assess the efficacy of EA at *Jing-jiaji* points (extra point; six points in three pairs, bilateral, 12.5 mm horizontally away from the corresponding cervical vertebra) for CS neck pain and the feasibility of a large clinical trial.

## Methods/Design

### Objective

The primary objective of this study is to evaluate the efficacy of EA intervention for CS neck pain.

### Design

**Figure 1:**
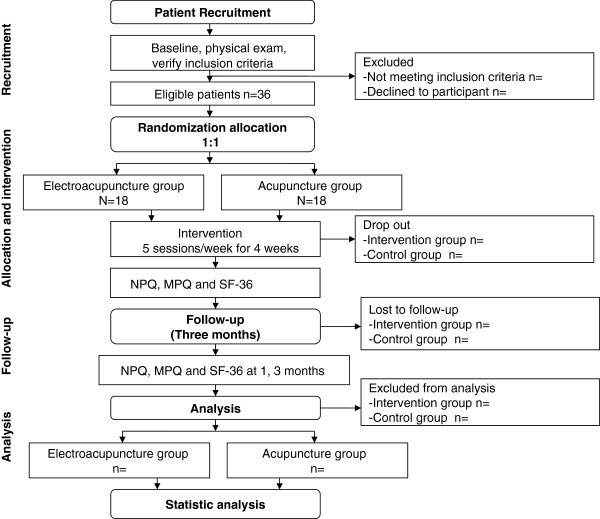
Flow chart of study process.

A randomized, assessor- and analyst-blinded, controlled trial to compare EA an group with an acupuncture group (Figure 1, Table 1) will be consisted in this study. This trial will be conducted at the Second Affiliated Hospital of Heilongjiang University of Traditional Chinese Medicine.

**Table 1:** Timing of visits and data collection

	**Week - 1**	**Week 0**	**Week 2**	**Week 4**	**1 month**	**3 months**
	**Baseline**	**Treatment phase**			**Follow-up phase (end-of-treatment)**	
Patients						
Informed consent	**×**					
Sign the informed consent		**×**				
Medical history	**×**					
Physical examination	**×**					
Randomization		**×**				
Intervention						
Electroacupuncture group (n = 18)		20 sessions of EA at *Jing-jiaji* points				
Comparisons						
Acupuncture group (n = 18)		20 sessions of acupuncture at *Jing-jiaji* points				
Outcomes^a^						
NPQ		**×**	**×**	**×**	**×**	**×**
MPQ		**×**	**×**	**×**	**×**	**×**
SF-36		**×**	**×**	**×**	**×**	**×**
Participants safety						
Adverse events		**×**	**×**	**×**	**×**	**×**

^**a**^NPQ, Northwick Park Neck Pain Questionnaire; MPQ, McGill Pain Questionnaire; SF-36, Short-Form 36 Scale.

This trial will include a four-week treatment period and a three-month follow-up period. After randomization, patients will receive 20-session treatments over a period of four weeks. Outcome measurements will be assessed at baseline (that is, one week after subjects are diagnosed with CS), as well as at the end of the second week and four weeks after randomization, and one and three months after conclusion of the treatment phase respectively. Patients will be informed that they may be assigned to an EA group, or an acupuncture group.

This trial will be carried out according to the principles of the Declaration of Helsinki (version Seoul 2008). This study protocol has been approved by the ethics review boards of the Second Affiliated Hospital of Heilongjiang University of Traditional Chinese Medicine, with permission number LLP2012029. All participants will be asked to provide written informed consent before enrollment having been given adequate time to consider what the trial involves. For subjects who decline to agree to participate in EA, other treatment options will be available.

### Randomization and allocation concealment

A randomization scheme will be conducted by the Evidence-Based Medicine Center of the Second Affiliated Hospital of Heilongjiang University of Traditional Chinese Medicine. According to a random sequence table, generated by SAS 8.2 software (SAS software (Beijing) Co., Ltd, 1 Changan Road, Dongcheng District, Beijing, China), participants who meet the inclusion criteria will be allocated randomly into an EA group and an acupuncture group in a 1:1 ratio. Two separate databases will be set up. One (participants’ database) includes basic information on issues such as name, contact details and so on, while the other (randomization database) holds data on patients registered in the trial and their allocation [25]. The randomization sequence schedule of group assignments and detailed treatments will be prepared by the Evidence-Based Medicine Center of the Second Affiliated Hospital of Heilongjiang University of Traditional Chinese Medicine and sealed in opaque envelopes. These sealed envelopes will be marked with the patients’ sequential numbers and kept by the screeners, who are not involved in the study.

### Blinding

The acupuncturists are in charge of sending randomization information and also receiving notices of the group assignation. As we know, it is not practical to blind the acupuncturists to treatment allocations and patients’ symptoms. In addition, it is not possible to prevent subjects from knowing whether they have received EA treatment or acupuncture intervention. However, it is feasible to blind outcome assessors and statisticians.

### Recruitment

Participants will be recruited via advertisements and posted notices. A data compilation form including the variables of interest will be completed by the corresponding researchers at each center. The obtained information will be recorded on an electronic database at each center, for subsequent statistical analysis.

## Eligibility

### Inclusion criteria

Participants will be included if they fulfilled the following criteria: (1) they are 18 to 75 years old (both male and female patients are included); (2) have a confirmed diagnosis of CS neck pain in accordance with the diagnostic criteria published by the Chinese Association of Rehabilitation Medicine (2010) [26] and with reference to the International Classification of Diseases, 10th edition (ICD-10) codes ( http://www.icd10data.com): M47.812 (other spondylosis without myelopathy or radiculopathy (cervical region)) [27]; (3) have a diagnosis of CS supported by a cervical radiographic examination, such as anteroposterior and lateral X-rays, or magnetic resonance imaging/computed tomography scans showing cervical spine degeneration or cervical disc herniation; (4) their main symptom is of neck pain with pain intensity of more than three points as measured on a visual analog scale (VAS) upon recruitment; (5) no acupuncture therapy or other relative treatment has been received within the last seven days prior to study entry; and (6) an informed consent document has been signed.

### Exclusion criteria

Patients with any of the following conditions will be excluded: (1) history of neck trauma, cervical fracture or cervical surgery, congenital spinal abnormality, or a diagnosis of systemic disease of the bones or joints of neck; (2) vertebral body or spinal canal cancer, tuberculosis, or severe osteoporosis; (3) complications of severe systematic diseases such as cardiocerebrovascular disease, tumors, diabetes mellitus, kidney disease, or digestive system disease; (4) pregnancy or lactation; (5) rejection or fear of acupuncture therapy; (6) use of any type of acupuncture within the seven days prior to study entry; and (7) use of any other treatments (drug or non-drug).

### Intervention

Intervention was designed according to record in the ancient book of *Yellow Emperor’s Inner Bible*[28] and according to recent studies of acupuncture as a treatment for CS. Subjects will receive acupuncture at bilateral acupuncture points *Jing-jiaji* (EX-B2.C4), *Jing-jiaji* (EX-B2.C5), and *Jing-jiaji* (EX-B2.C6). Sterile disposable acupuncture needles (40 mm in length and 0.30 mm in diameter; Andy brand, Guizhou Andy Medical Instrument Co., Ltd. ( http://www.andiyx.com; andiyx@163.com) will be perpendicularly inserted to a depth of 20 to 30 mm above the *Jing-jiaji* points in both groups. EA protocol was developed in consensus with acupuncturists and experts, who are good at acupuncture. In addition, this protocol is consistent with the STRICTA guidelines for the performance of EA studies [29].

### Acupuncture group

Acupuncture will be used in the acupuncture group. In this study, acupuncture at bilateral *Jing-jiaji* points for 30 minutes each time, five sessions a week for four weeks will be admininistered.

### Treatment group

In addition to normal acupuncture treatment, EA apparatus (Bio Medical™ Life Systems ( http://www.bmls.com; information@bmls.com)) will be connected to the needles for 30 minutes with interrupted waves (0.5Hz, 1 mA), five sessions a week for four weeks.

### Outcome measures

Three well-recognized patient-reported tools will be used to measure the outcomes. NPQ will be used as the primary outcome measure, and MPQ and SF-36 will be used as secondary outcome measures.

### Primary outcome

NPQ will be used to measure the symptoms of CS and function of the cervical vertebrae [30]. The tool with a higher score reflects a more serious disease state.

### Secondary outcome

The MPQ is a classic tool for assessing the intensity of neck pain, and a higher MPQ score reflects more serious pain [31]. The SF-36 is a general tool for measuring quality of life [32]. The tool consists of eight domains that measure quality of life of subjects in physical, mental and social dimensions. A higher score reflects a better quality of life.

## Statistical methods

### Sample size

This study is a pilot study for evaluation of the efficacy of EA on patients with CS and the feasibility of a large clinical trial. Due to the short duration, lasting four weeks, the desired sample size for this pilot study is 36 patients, with 18 for each group, assuming a drop-out rate of 20%, which is the minimum sample size necessary to evaluate the effectiveness of EA [33].

### Analysis

Data will be analyzed by a statistician blinded to allocation of groups. Statistical analyses will be conducted using SPSS 17.0 statistical software packages (Tel: (312) 651–3000), and the levels of significance will be reported at *P* < 0.05. The intention-to-treat (ITT) population was defined as the participants who are randomized and received at least one treatment session. The data analysis of baseline characteristics, as well as the primary and secondary outcomes is based on the ITT principle. In addition, analysis of covariance will also be conducted for possible baseline incomparability.

### Data handling

Investigators will enter the collected data required by the protocol into the case report forms. Non-obvious errors or omissions will be recorded on data query forms, which will be returned to the researchers’ workshop for resolution. The data from all centers will be pooled and summarized with respect to demographic baseline characteristics, effectiveness and safety observations.

### Patient safety

Any adverse experiences (known as unfavorable or unintended signs, or symptoms or disease occurring after treatment) related to EA treatment will be monitored. The research team will review all trial protocols, monitor subject safety, and investigate any adverse events. Trials will be terminated if there are concerns for patient safety.

### Quality control

All staff are required to undergo special training, including patient selection and exclusion, filling-up of the case report and acupuncture method, before participating in the trial. The monitors will check on case reports, and EA operation at the participating hospital twice a month. Drop-outs, withdrawals (and the reasons) from the study will be fully documented throughout the treatment and follow-up periods.

### Ethics

This trial is approved by the ethics review boards of the Second Affiliated Hospital of Heilongjiang University of Traditional Chinese Medicine.

## Discussion

CS neck pain remains a major public health problem. The results of this study will focus on neck pain caused by CS and will determine if EA is an effective intervention for this kind of symptom relief.

Acupuncture, one of the most important parts of traditional Chinese medicine, has been a form of health care in China for thousand years. It has been reported to have some benefits to CS patients, and also enhance their signs and symptoms.

Currently, no studies about EA treatment at *Jing-jiaji* points for patients with CS neck pain have been published; therefore, we designed this trial. This study aims to conduct a pilot study for a full scale trial of electroacupuncture at *Jing-jiaji* points for CS neck pain, and to look at its potential effectiveness in terms of CS neck pain of future episodes. Moreover, it also provides the feasibility of a large clinical trial. The data pooled will shed new light on acupuncture, especially for EA therapy for CS neck pain.

## Trial status

The trial is currently recruiting subjects.

## Abbreviations

ChiCTR: Chinese clinical trials register; CS: Cervical spondylosis; EA: Electroacupuncture; ITT: Intention-to-treat; MPQ: McGill pain questionnaire; NPQ: Northwick Park neck pain questionnaire; NSAID: Non-steroidal antiinflammtory; SF-36: Short-Form 36 scale.

## Competing interests

The authors declare that they have no competing interests.

## Authors’ contributions

JHY and QHZ contributed equally to this work. QHZ conceived the study and designed the study protocol. JHY drafted the manuscript. ZRS sought funding and ethical approval. All authors contributed to the further writing of the manuscript as well as read and approved the final manuscript.
